# Effect of the Application of Cold Working on the Aging Response and Mechanical Properties of an Al–4Cu–1.4Mg–0.6Ag Alloy

**DOI:** 10.1021/acsomega.6c06805

**Published:** 2026-10-07

**Authors:** Muhammad Farzik Ijaz, Talal Talib Alshammari, Mahmoud S. Soliman, Abdulrahman A. Alqarni, Hamad F. Alharbi

**Affiliations:** † Department of Mechanical Engineering, College of Engineering, 37850 King Saud University, P.O. Box 800, Riyadh 12372, Saudi Arabia; ‡ Mechanical Engineering Department, Jubail Industrial College, P.O. Box 10099, Jubail 31962, Saudi Arabia; § School of Metallurgy and Materials, University of Birmingham, Edgbaston, Birmingham B15 2TT, U.K.

## Abstract

This study investigates the effect of cold working on the aging response and mechanical properties of an Al–4Cu–1.4Mg–0.6Ag (wt %) alloy. The alloy was produced from pure elemental constituents and processed by casting, homogenization, hot rolling, and solution heat treatment. Following solution treatment, approximately 11% cold rolling was applied prior to artificial aging to produce a predeformed condition, while an undeformed condition was retained for comparison. The aging behavior of both conditions was evaluated as a function of aging time. Microstructural characterization was performed using optical microscopy, scanning electron microscopy (SEM), X-ray diffraction (XRD), and electron backscatter diffraction (EBSD). Cold working pronounced the deformation-induced dislocation density and promoted refinement of the second-phase precipitates. EBSD analysis revealed an increase in KAM-derived GND density in the predeformed condition, while SEM observations showed a finer precipitate distribution after aging. XRD analysis confirmed the formation and evolution of Al–Cu-rich and Al–Cu–Mg-containing secondary phases after the aging process. These findings highlight the role of the retained dislocation structure in stimulating heterogeneous precipitate nucleation and regulating precipitate refinement during aging. Finally, the predeformed condition exhibited a significant enhancement in mechanical properties after aging, with the yield strength increasing from 362 to 470 MPa and the ultimate tensile strength from 391 to 503 MPa, while the elongation increased from 2% to 5%. The improved mechanical response is associated with the dual effect of the deformation-induced dislocation structure and precipitate refinement during aging.

## Graphical abstract

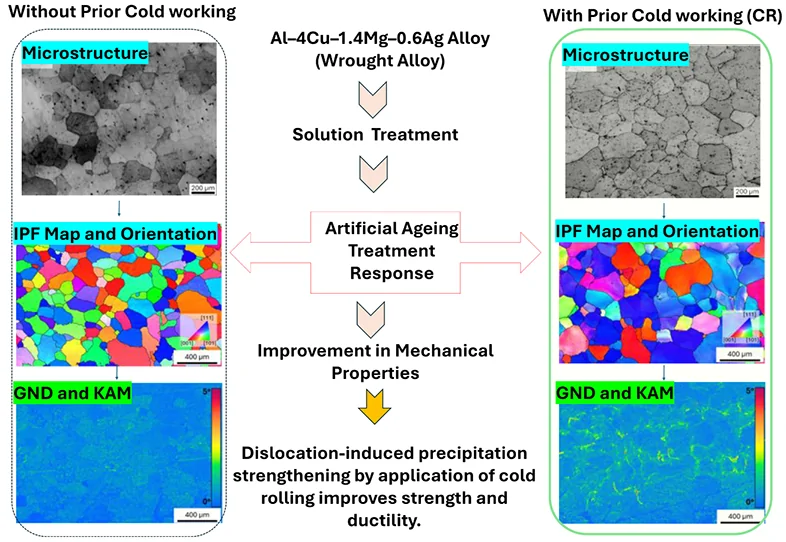

## Introduction

Aluminum alloys have earned widespread use across diverse industries due to their exceptional attributes, including low density, high corrosion resistance, thermal conductivity, and a favorable strength-to-weight ratio.^1–3^ Improving the mechanical properties of lightweight alloys holds the promise of broadening their applications while concurrently reducing operational costs through enhanced performance and decreased weight. Among the various Al alloys, Al–Cu–Mg–Ag has received substantial attention due to its remarkable thermal stability, rendering it particularly applicable in industrial applications.^4,5^ However, previous research predominantly centers on alloys with a high Cu/Mg ratio, where the Mg content is relatively low. This prompts us to explore the potential advantages associated with increasing the Mg content within Al–Cu alloys, such as the reduction of alloy density and the augmentation of the S phase, a crucial component comprising Al, Cu, and Mg.^6^ Furthermore, it is worth noting that Mg plays a pivotal role in governing the Cu/Mg ratio, typically ranging from 4 to 6 wt %for Cu and 0.6 to 2 wt % for Mg.^7^ However, our investigation reveals that decreasing the Cu/Mg ratio might not necessarily lead to an improvement in mechanical properties. The higher Mg content may increase the size of possible phases (S phase), negatively affecting the material’s mechanical properties, as indicated by previous research.^8–10^

Hence, our study delves into a potential approach to maintaining a substantial number of possible phases at smaller sizes (precipitate-strengthening phenomena). This strategy could enhance precipitate strengthening in the alloys.^11^ Additionally, predeformation may significantly increase dislocation density in the alloy matrix.^12^ These dislocations, in turn, could serve as nucleation sites for possible phases during the aging process.^13^

Predeformation (e.g., 5% uniform cold working) of Al alloys after solution treatment and before artificial aging generally accelerates the hardening response due to the formation of many nucleation sites for precipitates.^14^ In Al–Cu–Mg–Ag alloys with high Cu/Mg ratios, predeformation can decrease the density of the Ω phase due to the breaking of Mg–Ag coclusters known to act as nucleation sites for Ω precipitates. This reduction in the Ω phase is usually accompanied by an increase in the number density of the θ′ phase due to the increase in dislocation density, which promotes the heterogeneous nucleation of the θ′ phase.^7^ Liu et al.^14^ investigated the effects of predeformation on Al–5.3%Cu–0.8%Mg–0.5%Ag (high Cu/Mg ratio) by applying a 6% reduction in thickness before aging. The study showed that the hardness of the alloy increased due to the increase in dislocation density, which enhanced the nucleation of the θ′ phase. It was also noted that a predeformation of only 2.5% reduction did not show any improvement in the mechanical properties. Ringer et al.^15^ investigated the effect of predeformation before aging by applying a 6% cold-work reduction in thickness in an Al–5.3%Cu–0.4%Mg–0.4%Ag (high Cu/Mg ratio) alloy. The results showed that the density of Ω precipitates was reduced by about 35%, while θ′ precipitates increased, which decreased the hardness of the alloy from 145 HV to 135 HV. This is supported by Li et al.,^16^ who investigated the stability of Ω precipitates in an Al–5.1%Cu–0.8%Mg–0.5%Ag–0.7%Mn–0.13% Zr (high Cu/Mg ratio) alloy subjected to 5% predeformation during long exposure to high temperature after aging. The study revealed that the density of Ω precipitates decreased while S′ increased before it started to decrease again after 1000 h. Although the density of the Ω phase decreased, it was considered the major precipitate phase responsible for the hardening increase compared to the minor S′ precipitates. Bakavos et al.^17^ investigated the hardening of aluminum 2139 (Al–4.83%Cu–0.4%Mg–0.35%Ag–0.29%Mn) (high Cu/Mg ratio) under T8 treatment (cold worked before artificial aging ). It was found that predeformation enhanced the formation of θ′ and Ω phases, which increased the hardness of aluminum 2139. In another study, Bai et al.^18^ illustrated that the Ω precipitates in an Al–5.75%Cu–0.44%Mg–0.88%Ag–0.3%Mn (high Cu/Mg ratio) sheet were very sensitive to 1% stretching before aging at 180 °C. It was reported that preaging before the 1% stretching could significantly enhance the Mg–Ag coclusters, which provide large nucleation sites for the Ω phase and reduce the negative effect of dislocations on Ω precipitation. All the previous studies were applied to high Cu/Mg ratio alloys, where there is a limitation in applying predeformation to low Cu/Mg ratio alloys. Ying et al.^13^ applied a 5% prestrain to a low Cu/Mg alloy, resulting in improved mechanical performance, including yield, tensile strength, elongation, and resistance to fatigue crack growth, which was attributed to surface roughness-induced crack closure. This improvement is related to Cu–Mg clusters, where the S phase formed.

In this study, a higher predeformation level of 11% was selected, above the commonly reported 2–6% range, to investigate whether greater cold work can provide additional mechanical benefits. This strain level introduces significant dislocation density, accelerating aging kinetics and potentially enhancing the strength–ductility balance. The 11% strain remains within the practical limit for wrought Al alloys and avoids premature failure. This approach allows us to evaluate whether higher plastic deformation can further improve precipitation behavior and mechanical performance.

Although the mechanical behavior and microstructural evolution of Al–Cu–Mg–Ag alloys have been widely investigated, the combined effects of predeformation and aging in alloys with a low Cu/Mg ratio remain insufficiently explored. This is particularly important because increased Mg content can affect the size, distribution, and stability of the S phase. Accordingly, this study examines the influence of predeformation followed by different aging conditions, namely, the under-aged, peak-aged, and over-aged states, on the microstructure and mechanical properties of an Al–Cu–Mg-Ag alloy with a low Cu/Mg ratio.

## Experimental Work

The experimental procedure commenced with alloy casting, followed by a sequence of thermomechanical processing steps designed to produce specimens for the tensile and microstructural analyses. The detailed processing route consisted of homogenization, hot rolling, solid solution treatment, cold rolling, and artificial aging, as schematically illustrated in Figure 1A.

**Figure 1:**
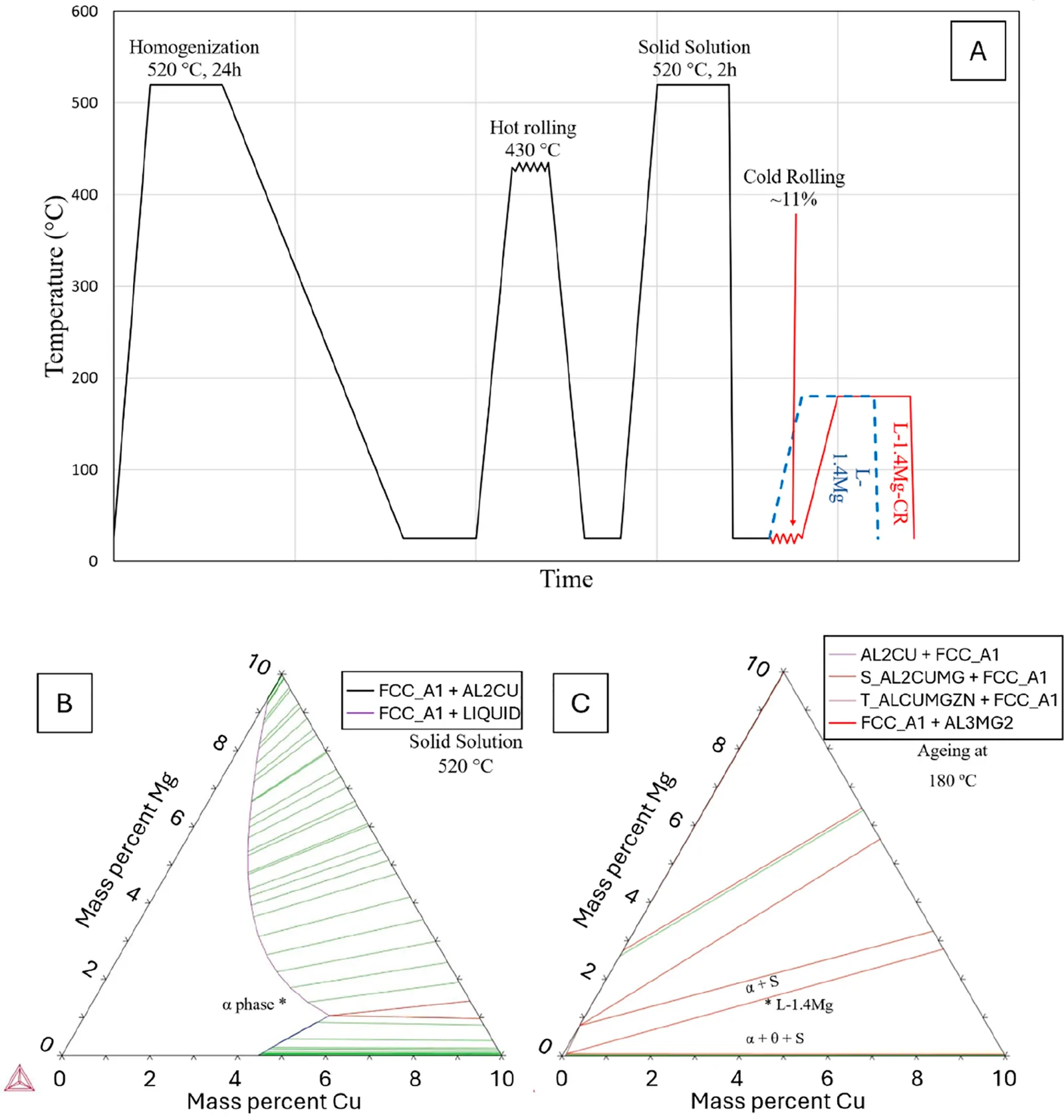
A) Schematic diagram of samples’ preparation process, B) materials simulation of the α phase, C) materials simulation of the S phase; B and C simulated by Thermo-Calc software.

As illustrated in Figure 1A, following casting, the produced ingot underwent a homogenization treatment to promote a uniform distribution of alloying elements. The as-cast ingot was heated to 520 °C and maintained at this temperature for 24 h. Upon completion of homogenization, the ingot was furnace-cooled to room temperature.^20^ The surface layer was subsequently removed to eliminate any oxide scale and casting-related defects.

The homogenized ingot, measuring 15 × 40 × 100 mm, was then subjected to hot rolling. Prior to rolling, the ingot was preheated to 430 °C for 2 h. Hot rolling was performed immediately after removal from the furnace, reducing the thickness from 15 mm to 2.5 mm, corresponding to an 85% reduction. The reduction was achieved through multiple passes with a thickness reduction of 0.1 mm per pass at an average rolling speed of 2.15 m/min. To maintain a stable processing temperature, the samples were reheated for 10 min between successive passes. A small thickness reduction of 0.1 mm per pass was selected because larger reductions per pass caused cracking and fracture of the specimens during preliminary rolling trials. Reheating between passes was therefore used to maintain sufficient workability throughout the large overall reduction in thickness.

Following hot rolling, the samples underwent solid-solution treatment at 520 °C for 2 h and were subsequently water-quenched. This treatment produced a supersaturated solid solution (SSSS), in which the alloying elements were dissolved within the α-Al matrix, as indicated by the phase diagram shown in Figure 1B.

To investigate the effect of predeformation on the aging response and mechanical properties, a portion of the solution-treated material was subjected to approximately 11% cold rolling prior to artificial aging. Cold rolling was carried out at a speed of 0.5 m/min with a thickness reduction of 0.05 mm per pass. The percentage of cold work was calculated from the reduction in sheet thickness according to the following equation:

1 $$CW\left(\%\right)=\frac{t_{0}-t_{f}}{t_{0}}\times 100$$

where *t*_0_ and *t*_*f*_ are the sheet thicknesses before and after cold rolling, respectively. The measured thickness reduction corresponded to approximately 11% cold work. The solution-treated material that did not undergo cold rolling was designated as the undeformed condition (L-1.4Mg), whereas the material subjected to approximately 11% cold rolling was designated as the predeformed condition (L-1.4Mg-CR), as summarized in Table 1.

**Table 1:** Nomenclature of the Developed Alloys Investigated in This Study Enlisted

	Compositions					
Sample name	Al	Cu	Mg	Ag	Cu/Mg ratio	% of predeformation (cold rolling)
L-1.4Mg	94.0	4.0	1.4	0.6	3	0
L-1.4Mg-CR	94.0	4.0	1.4	0.6	3	11

Following solution treatment and predeformation, the samples were artificially aged to promote the formation of the S phase, as predicted by Thermo-Calc simulations (Figure 1C). Artificial aging was carried out in a molten salt bath consisting of 50 wt % sodium nitrite (NaNO_2_) and 50 wt % potassium nitrate (KNO_3_). The salt mixture was heated to 180 °C and maintained until fully molten. Both the undeformed and predeformed samples were then immersed in the salt bath and aged at 180 °C for various durations to determine the peak-aged condition. Aging times of 10 min, 30 min, 1 h, 2 h, 4 h, 8 h, 10 h, 16 h, and 32 h were employed, after which the samples were water-quenched.

Specimens for microstructural examination were prepared following conventional metallographic practice. Grinding was performed on SiC abrasive papers of 500–1200 grit, after which the surfaces were finished with a 0.05 μm alumina suspension. Etching for 30 s in etchant No. 3 of ASTM E407-99 (2 ml HF, 3 ml HCl, 5 ml HNO_3_, 200 ml H_2_O) then made the grain structure and the microstructural constituents visible. Optical microscopy was performed using an Olympus microscope to examine the grain morphology. A JXA-840A electron probe microanalyzer (JEOL, Japan) served for scanning electron microscopy (SEM), whereas the constituent phases were identified by X-ray diffraction (XRD) in a MAXima XRD-7000 diffractometer operating with Cu Kα radiation. Grain morphology, crystallographic orientation, and local lattice misorientation were assessed by electron backscatter diffraction (EBSD). Each EBSD map covered 125,538 μm^2^ and was collected with 0.5 μm steps, the beam being set to 20 kV and the working distance fixed at 15 mm.

Mechanical characterization was performed following artificial aging. Vickers hardness measurements were conducted using a Wilson Instruments WOLPERT UH930 hardness tester with a load of 10 kgf and a dwell time of 15 s. The reported hardness values represent the average of five indentations obtained from different locations on each specimen. Tensile specimens were machined from the solution-treated material according to ASTM E8/E8M standards using electrical discharge machining (EDM) and subsequently aged to the peak-aged condition determined from the hardness measurements. Tensile testing was carried out at room temperature using an EPSILON extensometer at an initial strain rate of approximately 10^–3^ s^–1^. At least five specimens were tested for each condition to ensure the reproducibility of the results.

## Experimental Results

Table 2 shows the chemical composition analysis using the SPECTROMAXx metal analyzer for all samples tested in this study. It is clear that the measured chemical compositions of all samples are within the acceptable range compared to the designed experiments discussed in the previous chapter (see Table 1). Table 2 shows that the measured Cu/Mg ratio of alloy L-1.4Mg is 2.96 compared to the target of 3.

**Table 2:** Chemical Composition Analysis by SPECTROMAXx Metal Analyzer of the Tested Samples

	Composition				
Samples	Al	Cu	Mg	Ag	Cu/Mg ratio
L-1.4Mg	93.73	4.23	1.43	0.61	2.96

The microstructures of the undeformed and predeformed alloys were examined in the peak-aged condition. As shown in Figure 2(a,b), the application of approximately 11% cold rolling prior to aging resulted in only a slight increase in the average grain size, from 139 μm in the undeformed sample to 152 μm in the predeformed sample. This relatively small change suggests that the influence of predeformation on grain structure was minimal and that grain refinement did not contribute significantly to the observed strengthening.

**Figure 2:**
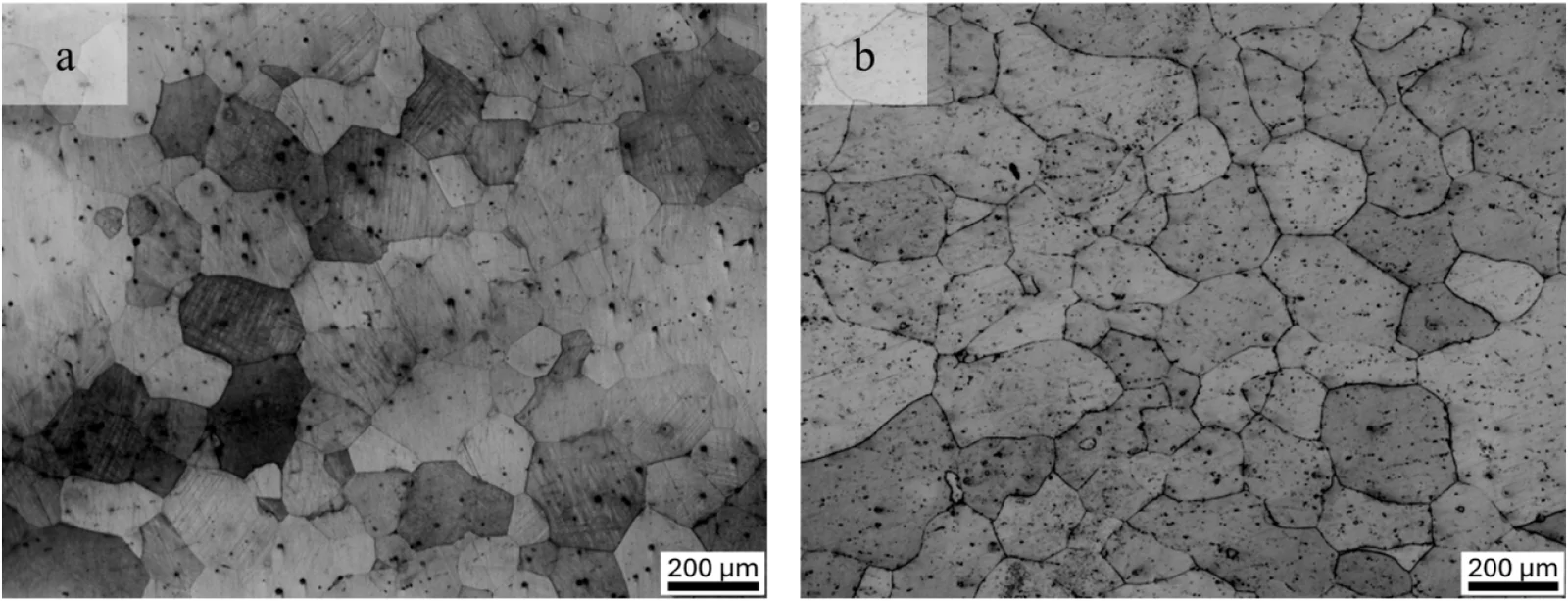
Optical micrographs of a) L-1.4Mg alloy and b) L-1.4Mg-CR alloy under peak-aged conditions.

In contrast, predeformation had a pronounced effect on the precipitation characteristics of the alloy. Figure 3(a) shows that the undeformed alloy (L-1.4Mg) contained second-phase precipitates with an average size of 18 μm and a volume fraction of 0.51%, distributed relatively uniformly throughout the microstructure. Following the application of cold rolling prior to aging, the average precipitate size decreased substantially to approximately 5 μm in the L-1.4Mg-CR alloy, while maintaining a comparable volume fraction of 0.44%, as shown in Figure 3(b). These observations indicate that a relatively simple cold deformation treatment can effectively regulate the precipitation behavior of the alloy by promoting a finer precipitate distribution. The refinement of the precipitates is attributed to the deformation-induced defects introduced during cold rolling, which provide additional heterogeneous nucleation sites during aging. Consequently, predeformation influences the precipitation response more significantly than the grain structure, resulting in enhanced precipitation strengthening and improved mechanical performance.

**Figure 3:**
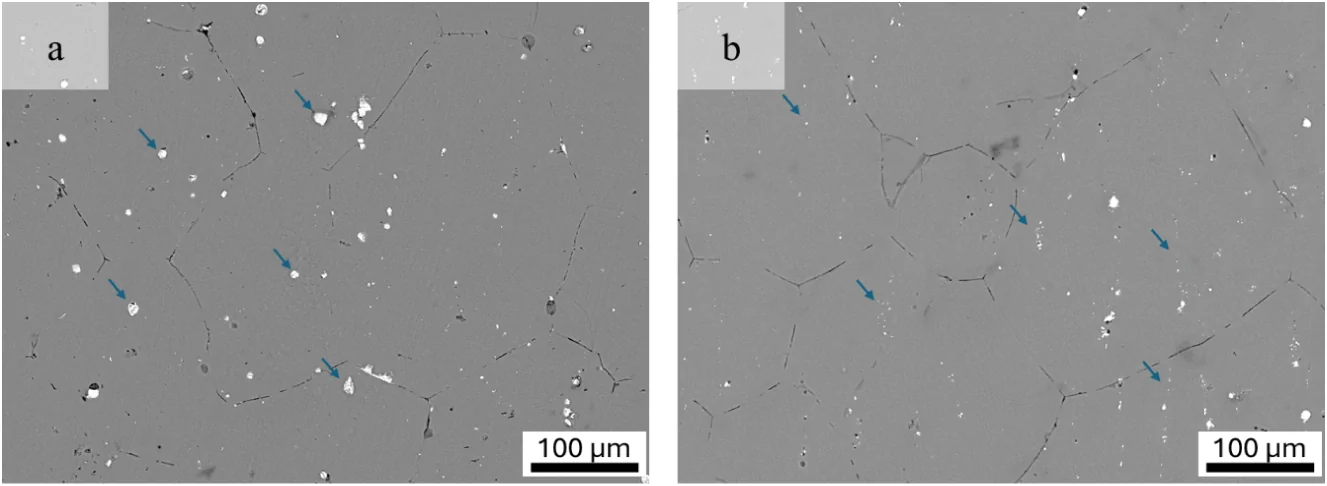
SEM analysis of the low Cu/Mg ratio: a) L-1.4Mg and b) L-1.4Mg-CR.

To identify the precipitates formed during aging, XRD analysis was performed on the peak-aged alloy, as shown in Figure 4. The diffraction peaks located at approximately 38.5°, 44.7°, and 65.1° correspond to the α–Al matrix and are indexed to the (111), (200), and (220) crystallographic planes, respectively. In addition to the matrix reflections, several low-intensity peaks associated with the S phase (Al_2_CuMg) were detected, confirming the formation of this strengthening phase during artificial ageing. A weak diffraction peak corresponding to the θ phase was also observed, indicating the presence of a minor amount of Al_2_Cu-based precipitates. The XRD results therefore corroborate the microstructural observations and suggest that the refined precipitate structure observed in the predeformed condition is predominantly associated with the formation of S-phase precipitates. The substantial reduction in precipitate size, together with the increased density of deformation-induced defects, is believed to be advantageous for enhanced precipitation strengthening and the resulting significant improvement in the mechanical properties of the alloy.

**Figure 4:**
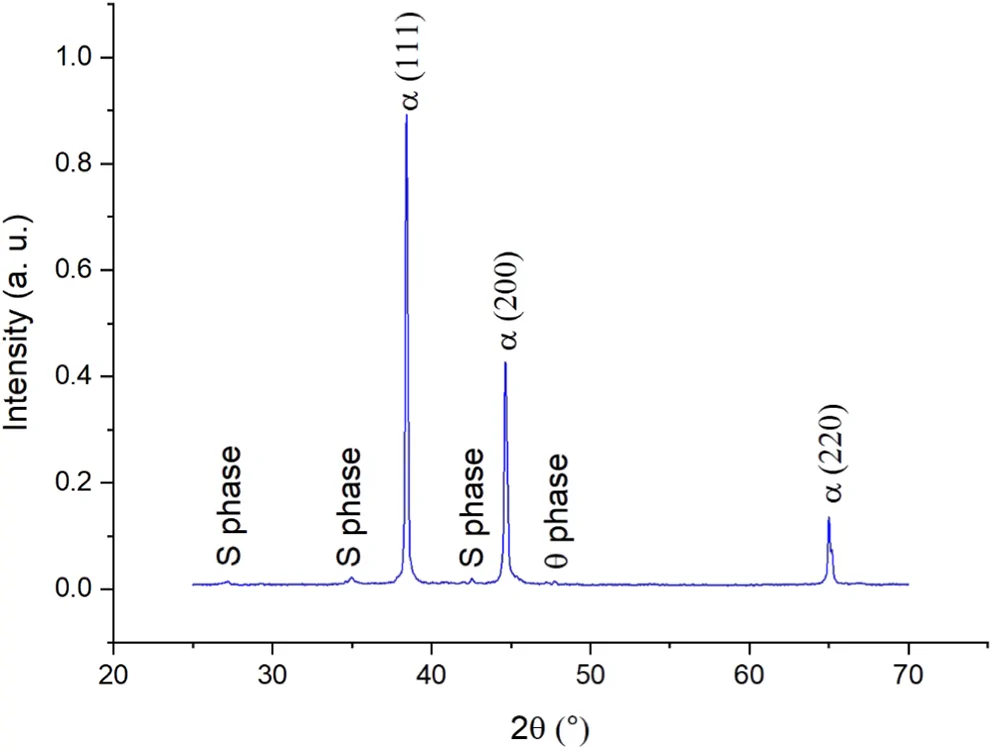
XRD pattern of L-1.4Mg showing the possible phases.

To corroborate the XRD results identifying the formation of secondary phases within the Al matrix, SEM–EDS analysis was performed to investigate the chemical composition of selected second-phase precipitates. Figure 5(a–b) presents SEM images and corresponding EDS spectra obtained from two representative areas selected for compositional analysis. Indeed, as shown in Figure 5(a), the second-phase particle exhibited an Al–Cu-rich composition, containing 58.49 wt % Al and 41.51 wt % Cu, indicating an Al–Cu-rich second-phase constituent. On the other hand, Figure 5(b) revealed an Al–Cu–Mg–Ag-containing precipitate mainly consisting of 54.85 wt % Al, 32.61 wt % Cu, 7.42 wt % Mg, and 5.12 wt % Ag. The simultaneous presence of Cu and Mg with minor Ag is consistent with an S-phase-related Al_2_CuMg precipitate containing Ag.^7,10^

**Figure 5:**
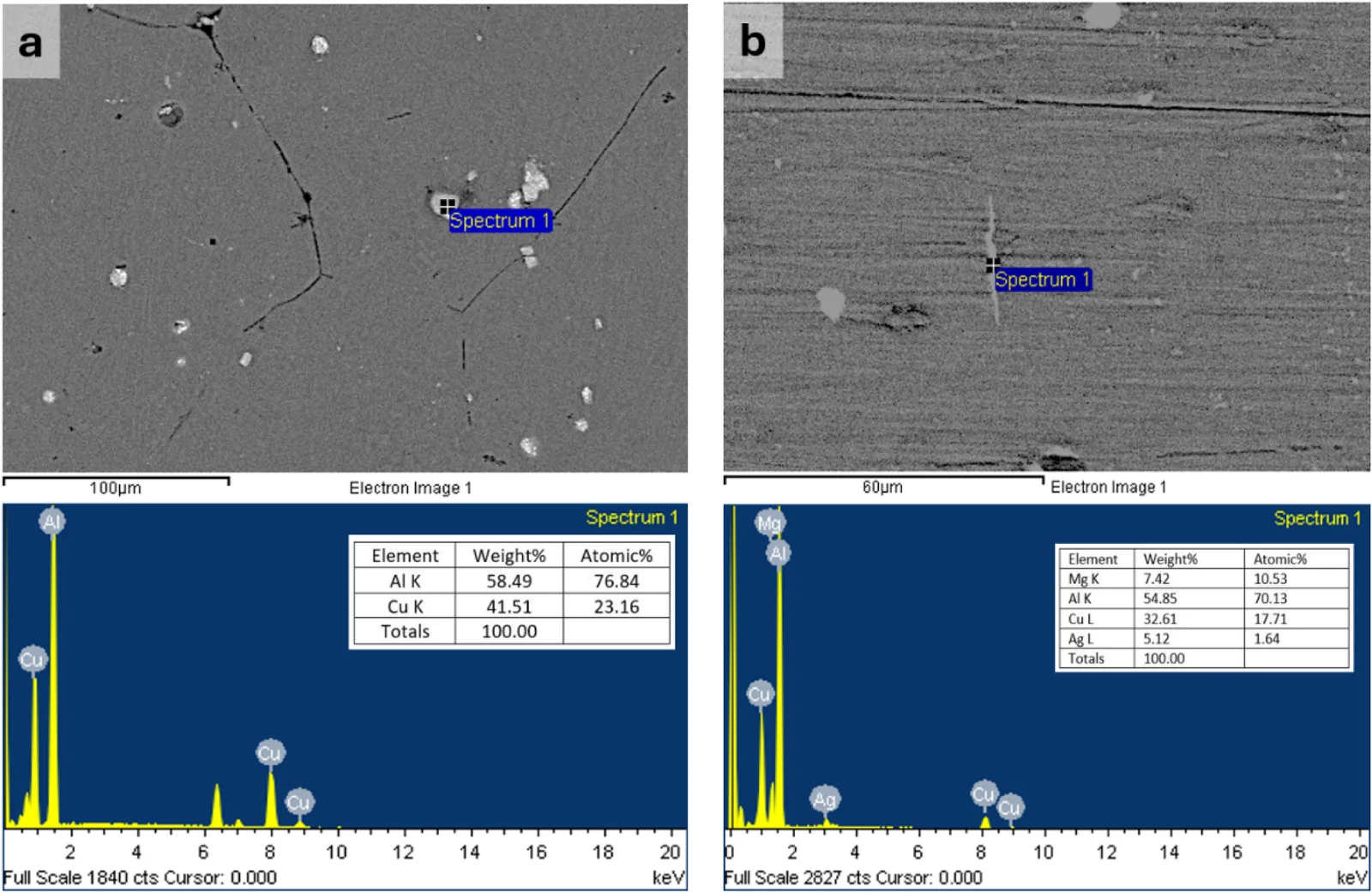
SEM–EDS characterization of second-phase precipitates in the Al–4Cu–1.4Mg–0.6Ag alloy: (a) Al–Cu-rich precipitate and (b) S-phase (Al_2_CuMg)-containing precipitate with minor Ag.

The microstructural evolution of the alloys was further investigated using EBSD analysis in the peak-aged condition. Figure 6 presents the inverse pole figure (IPF) and kernel average misorientation (KAM) maps for both the undeformed (L-1.4Mg) and predeformed (L-1.4Mg-CR) samples. The IPF maps (Figure 6a,b) reveal predominantly equiaxed grains, indeed no evidence of a pronounced crystallographic texture was observed. Interestingly, the average grain size increased slightly from 139 μm in the undeformed sample to 152 μm in the predeformed sample, which is consistent with the optical microscopy observations as shown in Figure 2. The slight increase in grain size may be associated with recovery processes occurring during artificial aging, which can reduce the stored deformation energy introduced by cold rolling and promote limited grain boundary migration.^21^

**Figure 6:**
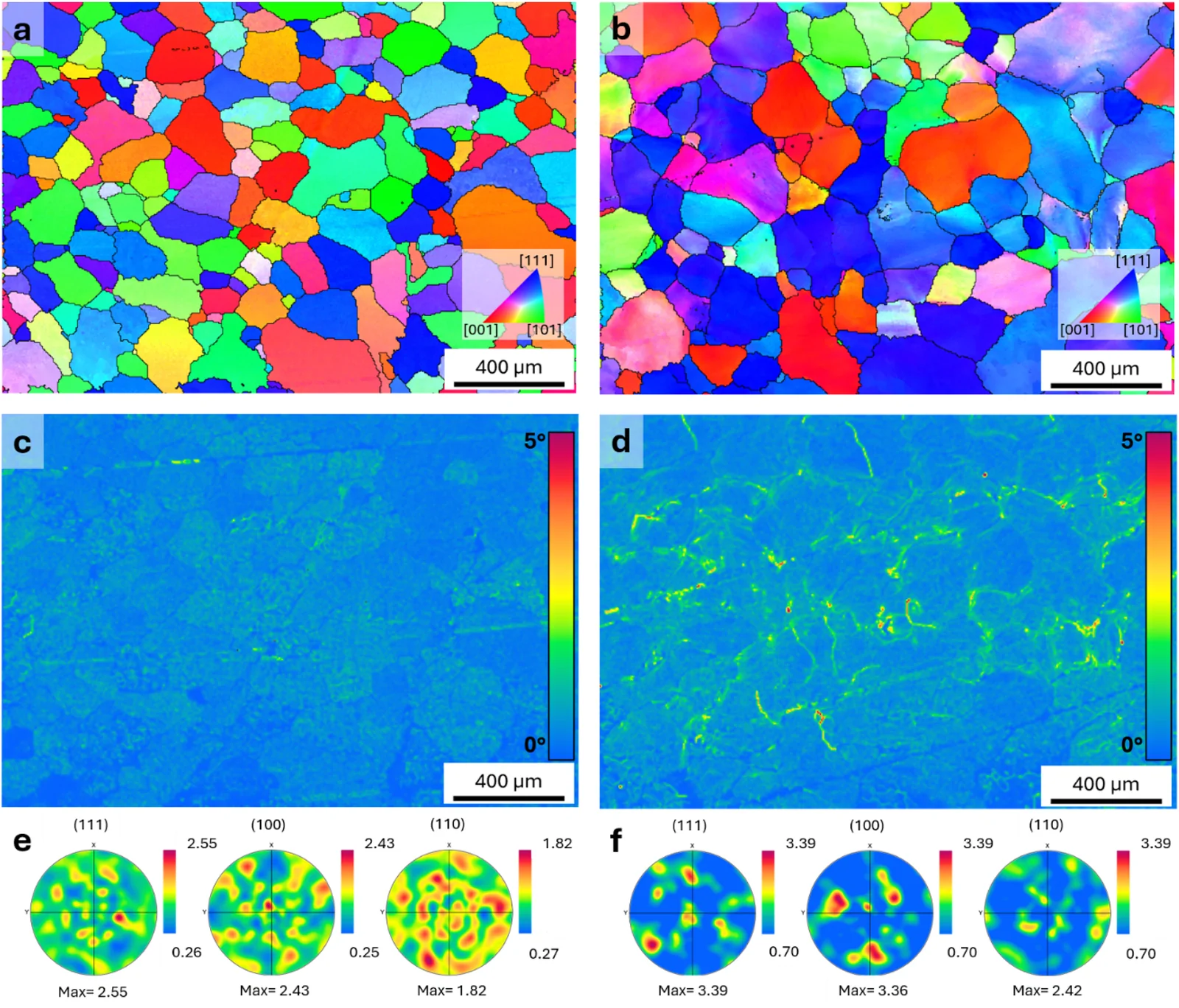
EBSD analysis of the peak-aged Al–Cu–Mg–Ag alloy: (a) IPF map of L-1.4Mg, (b) IPF map of L-1.4Mg-CR, (c) KAM map of L-1.4Mg, (d) KAM map of L-1.4Mg-CR, (e) pole figures of L-1.4Mg, and (f) pole figures of L-1.4Mg-CR.

This slight increase in grain size indicates that the enhanced mechanical properties of the predeformed alloy cannot be attributed to grain-refinement strengthening. The KAM maps (Figure 6c,d) provide insight into the local lattice misorientation and strain distribution within the microstructure. In both samples, elevated KAM values are primarily concentrated near grain boundaries, indicating the presence of local strain gradients and geometrically necessary dislocations (GNDs). However, the predeformed sample exhibits a greater density of localized misorientation regions compared with the undeformed condition, suggesting the retention of deformation-induced dislocation structures following the 11% cold rolling process and subsequent aging treatment.

The corresponding pole figures in Figure 6(e,f) indicate a relatively weak crystallographic texture under both conditions, with a modest increase in texture intensity after predeformation. The maximum intensities increased from 2.55, 2.43, and 1.82 m.r.d. in the undeformed condition to 3.39, 3.36, and 2.42 m.r.d. in the predeformed condition, respectively. Using the KAM data in Figure 6(c,d), acquired with a step size of 0.5 μm, the estimated GND density increased from 5.73 × 10^13^ m^–2^ in the undeformed condition to 7.69 × 10^13^ m^–2^ after predeformation, indicating greater EBSD-resolvable lattice curvature. However, KAM analysis does not directly resolve individual dislocations or precipitate nucleation events; therefore, the calculated GND densities should be considered comparative estimates rather than absolute dislocation densities. The finer second-phase distribution observed by SEM is consistent with heterogeneous precipitation promoted by deformation-induced defects, as reported in previous studies. Nevertheless, direct confirmation of this mechanism would require higher-resolution characterization, such as TEM.

Figure 7 shows the improvement in the hardness of the predeformed L-1.4Mg-CR samples, which were harder than the undeformed ones during the entire aging span. The underaged samples showed an ∼10 HV difference between the undeformed L-1.4Mg and the deformed L-1.4Mg-CR ones. This increment in hardness progressed until it reached a peak point. Both the deformed and undeformed alloys reached peak hardness at the same time and maintained it during the over-aging stage. Based on the age-hardening curves, the peak-aged condition for both L-1.4Mg and L-1.4Mg-CR was obtained after aging at 180 °C for 4 h. The tensile tests revealed an excellent improvement in the mechanical properties of the deformed samples (L-1.4Mg-CR). In particular, the ultimate tensile strength increased from 391 to 503 MPa by 24%, the yield strength increased from 362 to 470 MPa by 30%, and the strain at failure (elongation %) increased from 0.02 (2%) to 0.05 (5%) by 150%, as reported in Figure 8.

**Figure 7:**
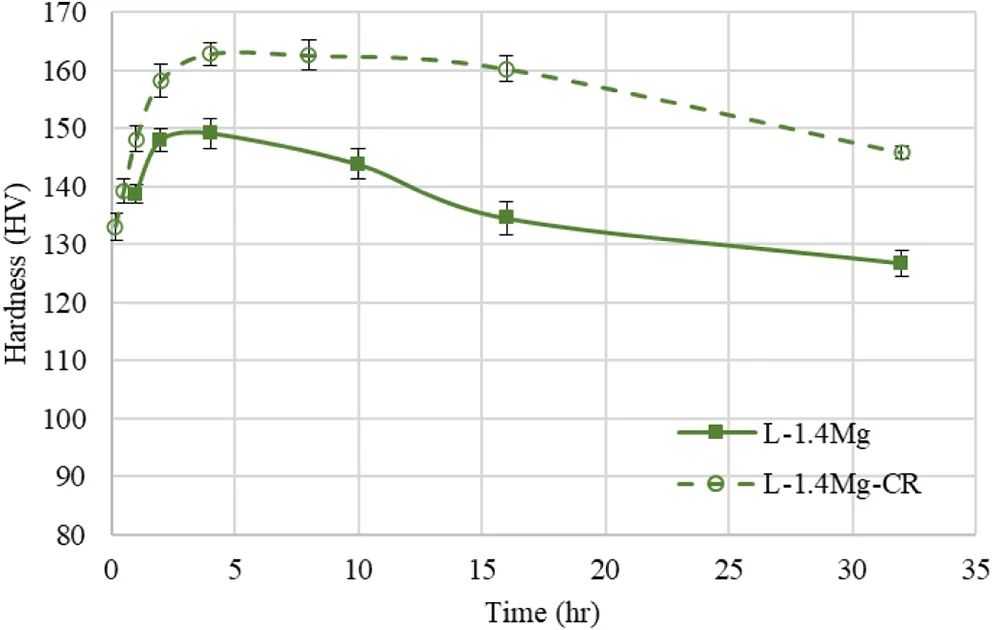
Comparative analysis of age-hardening curves for L-1.4Mg alloy with and without predeformation.

**Figure 8:**
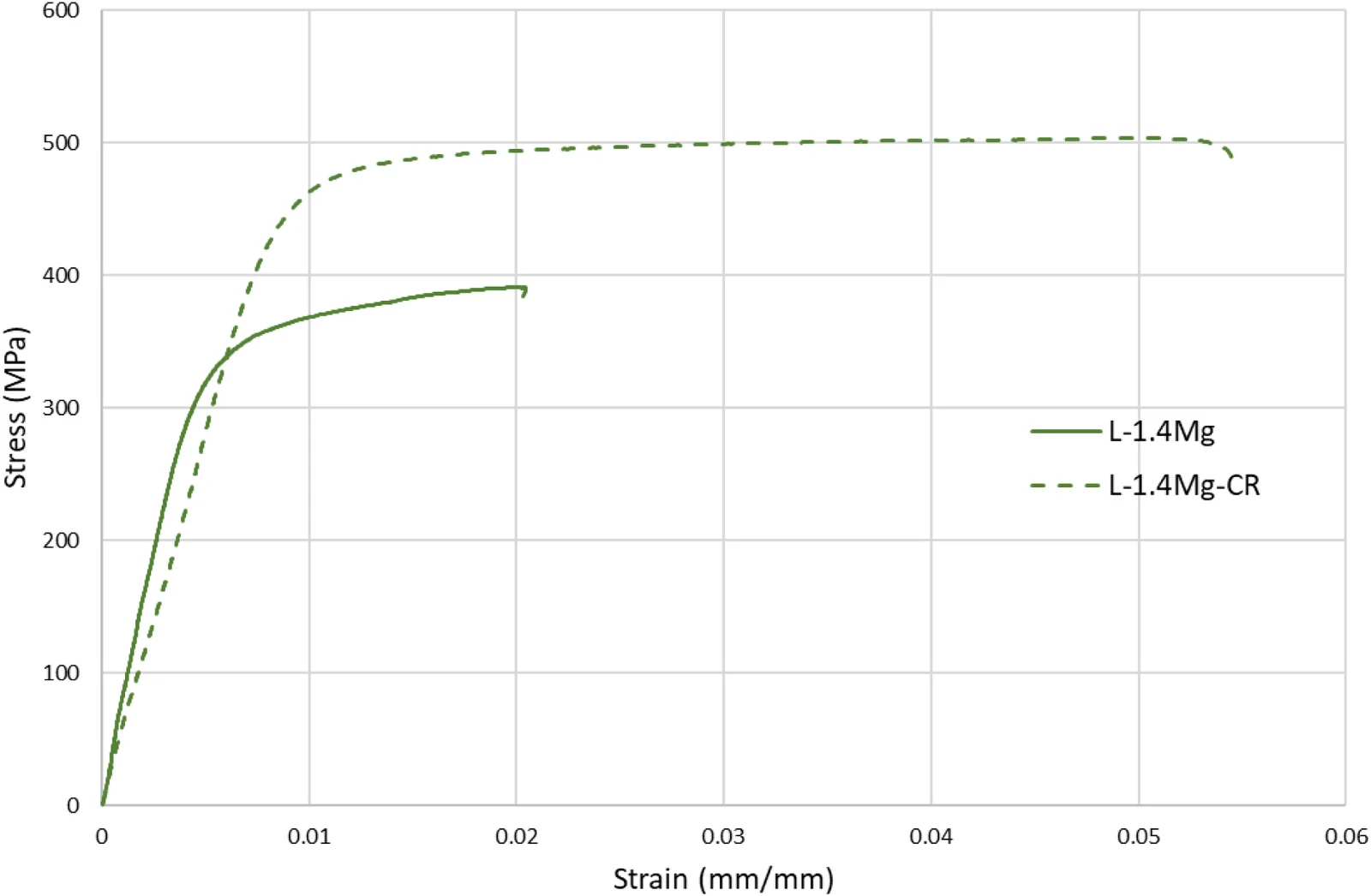
Tensile test results demonstrating the influence of predeformation on a low Cu/Mg Alloy under peak-aged conditions.

## Discussion

A comparison was made between the microstructure and mechanical properties of the two samples. Table 3 shows that the grain size in both samples was insignificantly altered. This may have been due to the aging process being conducted at a temperature that was not high enough to cause grain recrystallization. However, the particle size in the predeformed samples (L-1.4Mg-CR) decreased significantly from the undeformed sample (L-1.4Mg). This was likely due to an increase in the dislocation density caused by the cold rolling process. The high dislocation density increased the nucleation sites of particles, as reported by previous studies^14,15^ and supported by SEM images of the deformed sample. The particles were formed in different sizes on line arrangement, such as necklace-like granules. This arrangement suggests that the dislocation lines might be the best sites to form the phase clusters.

**Table 3:** Effect of Predeformation in a Low Cu/Mg Ratio at Peak-Aged Condition

Alloy	Yield stress (MPa)	Tensile stress (MPa)	% of fracture strain	Avg. hardness (HV)10	Avg. grain size (μm)	Avg. particle size (μm)	Avg. peak-aging time (h)
L-0.6Ag	362 ± 7	391 ± 12	2	149	139	5.3	4
L-0.6Ag-CR	470 ± 4	503 ± 3	5	163	152	1.7	4
Changes	↑ 30%	↑ 29%	↑ 150%	↑ 9%	↑ 9%	↓ 58%	-

The used composition may have four phases, consisting of the α phase, θ phase, and S phase, depending on Thermo-Calc simulation software (Figure 1) and XRD results (Figure 4), and the Ω phase depending on literature.^22^ SEM analysis showed an increase in the number of θ and S phases while their size decreased. Previous TEM studies have shown that S-phase (Al_2_CuMg) precipitates in low Cu/Mg Al–Cu–Mg alloys generally exhibit a lath-like morphology, with their long axis approximately parallel to ref 23. In contrast, θ′-type Al_2_Cu precipitates typically exhibit a plate-like morphology on planes.^10^ Predeformation may alter their size and distribution by promoting heterogeneous precipitation at deformation-induced defects. However, the present SEM and XRD analyses cannot directly resolve the nanoscale morphology or coherency of these precipitates; therefore, these characteristics are discussed based on previous TEM observations. This positively impacted the mechanical performance, as supported by the hardness curve in Figure 7 and the stress–strain curve in Figure 8. However, the improvement in mechanical performance may have been entirely dependent on precipitate strengthening, where the solid-solution strengthening has a low effect due to the aging process, where most of the atoms may cluster to form precipitates. These precipitates might be at a high coherency level, where the hardness was at the peak of the aging curve. The increase in elongation after predeformation and aging is unlikely to result solely from a reduction in dislocation strengthening. The finer and more uniformly distributed precipitates may also contribute by reducing localized strain accumulation and promoting more homogeneous plastic deformation, thereby delaying strain localization and necking. Together with partial recovery of the deformation-induced dislocation structure during aging, this may explain the improved strength–ductility balance observed in the predeformed condition.

Additionally, the grain-size strengthening was calculated by the Hall–Petch equation:^24^

2 $$\Delta \sigma_{Gb}=k\left(d^{-1/2}\right)$$

The Hall–Petch slope of Al4Cu alloys (0.08 MPa m^1/2^) is denoted by *k* and *d* represents the diameter of the grain size, where the results of both samples were insignificant, with only 6.4 and 6.7 MPa recorded for the deformed and nondeformed samples, respectively.

To evaluate the dislocation strengthening mechanism, EBSD-KAM analysis was performed, and the GND density was estimated using the following equation:^25^

3 $$\rho_{GND}=\frac{\theta }{b\times L}$$

where θ is the average misorientation angle in radians, *b* is the Burgers vector for aluminum (2.86 × 10^–10^), and *L* is the EBSD step size. Based on the high-magnification KAM map (step size = 0.5 μm), the KAM-derived GND density increased from 5.73 × 10^13^ m^–2^ in the undeformed sample to 7.69 × 10^13^ m^–2^ in the predeformed sample. The contribution of dislocation density to yield strength was then calculated using the classical Taylor equation:^25^

4 $$\Delta \sigma =M\times \alpha \times G\times b\times \sqrt{\rho_{GND}}$$

where *M* = 3.06 is the Taylor factor for FCC aluminum,α = 2 is a material constant, *G* = 26 × 10^9^ Pa is the shear modulus of aluminum, and *b* is the Burgers vector. The calculation revealed a dislocation strengthening contribution of approximately 54 MPa due to the 11% predeformation by cold rolling. This moderate increase confirms that dislocations contributed to the alloy’s strength, one of the main strengthening mechanisms. The KAM-derived GND density should be regarded as a comparative estimate, since the 0.5 μm EBSD step size may not resolve fine-scale dislocation structures. In addition, KAM primarily reflects geometrically necessary dislocations associated with measurable lattice curvature and does not represent the total dislocation density. Direct characterization of finer dislocation structures would require TEM analysis. In addition, the dislocations played a more critical role in providing nucleation sites for the S-phase precipitates. This indirect influence is evidenced by the finer precipitates observed in the predeformed sample. As the contribution of precipitate strengthening significantly increases, the Orowan equation is used to calculate the S-phase contribution to the yield:^26^

5 $$\Delta \sigma_{p}=\frac{0.13\times G\times b}{\lambda }\times \ln\left(\frac{2r}{b}\right)$$

where *r* and λ are the average particle radius and spacing, respectively. The average particle radius was estimated using $r=\sqrt{A/\pi }$ . The interparticle spacing λ was calculated as $\lambda =2r/\sqrt{3f}$ . The measured particle characteristics and the corresponding calculated precipitation-strengthening contributions are summarized in Table 4. The results showed a strengthening contribution of approximately 15 MPa for the nondeformed alloy and 25 MPa for the predeformed alloy. This moderate increase confirms that although the measured particle area fraction decreased slightly in the predeformed condition, the finer particle size and closer spacing improved the efficiency of precipitation hardening. This suggests that predeformation promotes the formation of smaller, more effective S-phase precipitates, enhancing the mechanical properties of the alloy.

**Table 4:** Measured Particle Characteristics and Calculated Precipitation-Strengthening Contribution for the L-0.6Ag and L-0.6Ag-CR Conditions

Alloy	Average particle size, *d*_*p*_ (μm)	Particle area fraction, *f* (%)	Interparticle spacing, λ (μm)	Precipitation strengthening, Δσ_*p*_ (MPa)
L-0.6Ag	5.27	0.798	5.23	15
L-0.6Ag-CR	1.67	0.628	3.19	25

The Taylor and Orowan calculations provide approximate strengthening contributions rather than a strict separation of independent mechanisms, since predeformation affects both dislocation density and subsequent precipitation. Dislocations introduced by cold working can act as heterogeneous nucleation sites for S-phase precipitates, thereby modifying their size and spacing.^27^ The interaction of dislocations with precipitates depends on precipitate size, coherency, and morphology; fine shearable precipitates may be cut by dislocations, whereas larger or nonshearable S-phase precipitates are generally bypassed by Orowan looping.^28^ Similar transitions between shearing and bypassing have been reported in Al–Cu–Mg–Ag alloys.^28^ Since the present SEM and XRD analyses cannot directly resolve these nanoscale interactions, the Orowan calculation should be regarded as an approximate estimate of precipitation strengthening.

The calculated strengthening contributions are lower than the experimentally observed increase in yield strength, which may arise from limitations in the microstructural parameters used in the models. The Taylor calculation is based on the KAM-derived GND density and, therefore, does not account for the total dislocation population. Similarly, the Orowan calculation is based on precipitate dimensions obtained from SEM, which may not fully represent the finer strengthening precipitates formed during aging. Consequently, the calculated values should be considered approximate and may underestimate the actual contributions from dislocations and precipitation strengthening. Solid-solution strengthening may also contribute to the overall strength, although its individual contribution cannot be quantified from the present measurements.

The stress–strain curve showed significant improvements in yield strength, ultimate tensile strength, and elongation. Also the hardness with aging time was improved for all periods of time investigated. The present study reached 11% of predeformation, which was higher than the 6% reported in the literature, as compared in Table 5.

**Table 5:** Summary of Previous Research on Predeformation

Alloy (wt %)	Cu/Mg ratio	% of predeformation	Yield stress (MPa)	Refs
Al–4Cu–1.4Mg–0.6Ag	3 (low)	11	470	Present study
Al–3.6Cu–1.6Mg	2.3 (low)	5	362	^14^
Al–5.3Cu–0.8Mg–0.5Ag	6.6 (high)	6	451	^15^
Al–5.3Cu–0.4Mg–0.4Ag	13.3 (high)	6	-	^16^
Al–5.1Cu–0.8Mg–0.5Ag–0.7Mn–0.13Zr	12.8 (high)	5	-	^17^
Al–4.83Cu–0.40Mg–0.35Ag–0.29Mn	12 (high)	1–2	-	^18^
Al–5.74Cu–0.44Mg–0.88Ag–0.3Mn	13 (high)	1	458	^19^

Nonetheless, the application of 11% cold rolling prior to aging is expected to increase the dislocation density within the supersaturated solid solution (SSSS). These deformation-induced dislocations may act as additional heterogeneous nucleation sites during subsequent aging, thereby influencing the precipitation behavior of the alloy. As schematically illustrated in Figure 9, it is plausible that the increased dislocation density promotes a finer and more homogeneous distribution of precipitates compared with the nondeformed condition. Such a precipitation structure is expected to reduce the interparticle spacing and increase the resistance to dislocation motion during mechanical loading. Consequently, the observed increase in yield strength following predeformation may be attributed to the combined effects of strain hardening and a potentially enhanced precipitation response associated with the higher dislocation density. Although direct microstructural evidence is not available in the present study, this proposed mechanism is consistent with the observed mechanical behavior. Detailed characterization of the precipitation features and their interaction with deformation-induced dislocations will be investigated in future work by using transmission electron microscopy (TEM).

**Figure 9:**
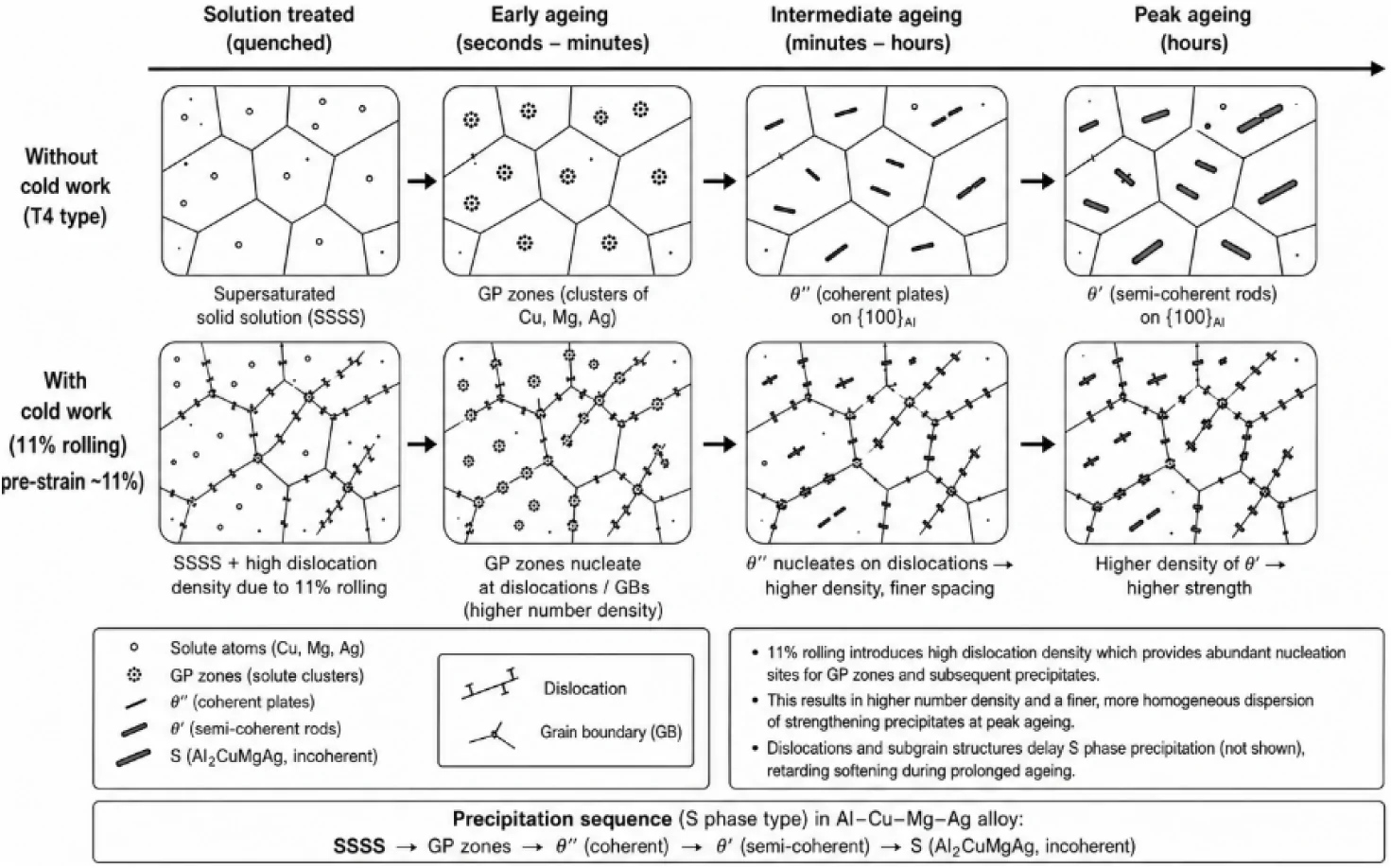
Schematic indicating the precipitation evolution in a low Cu/Mg-ratio Al–Cu–Mg–Ag alloy during ageing with and without cold work . (Graphic layout and conceptual design developed with the assistance of Google Gemini and ChatGPT GPT-5.6 Luna.)

## Conclusions

The SEM and EBSD results revealed a synergistic relationship between the deformation-induced dislocation structure and precipitate refinement. For the cold-rolled specimen, the KAM-derived GND density, while the grain size changed only slightly. The higher dislocation density provided additional sites for precipitate nucleation and promoted a finer second-phase precipitate distribution during aging.
XRD confirmed the formation and evolution of secondary phases during ageing, while SEM–EDS revealed Al–Cu-rich and Al–Cu–Mg–Ag-containing precipitates. The latter composition was consistent with an S-phase-related Al_2_CuMg precipitate containing a small amount of Ag.
The cold-rolled specimen showed higher peak hardness and a stronger aging response than the noncold-rolled condition. This indicates that predeformation influenced the precipitation process and promoted precipitate refinement during aging.
The improved mechanical response resulted from the combined effect of the retained dislocation structure and precipitate refinement. The yield strength increased from 362 to 470 MPa, and the ultimate tensile strength increased from 391 to 503 MPa, while the elongation increased from 2% to 5%.
5. The higher dislocation density and finer precipitate distribution played a significant role in increasing the resistance to dislocation motion and enhancing the mechanical properties of the investigated alloy.

## Data Availability

The datasets used and/or analyzed during the current study are available from the corresponding author on reasonable request.
